# Supplementary material for: Cryo-EM structures of amyloid-β filaments with the Arctic mutation (E22G) from human and mouse brains
Source: Acta Neuropathol. 2023 Jan 7;145(3):325–33. doi: 10.1007/s00401-022-02533-1 (PMC9925504; doi:10.1007/s00401-022-02533-1)
Supplement: Supplementary file 1 — Supplementary file1 (PDF 455 kb) [file 401_2022_2533_MOESM1_ESM.pdf]

CRYO-EM STRUCTURES OF AMYLOID-β FILAMENTS WITH THE ARCTIC MUTATION (E22G) FROM HUMAN AND MOUSE BRAINS

Yang Yang[1], Wenjuan Zhang[1±], Alexey G. Murzin[1], Manuel Schweighauser[1], Melissa Huang[1☓], Sofia Lövestam[1], Sew Y. Peak-Chew[1], Takashi Saito[2+], Takaomi C. Saido[2], Jennifer Macdonald[1], Isabelle Lavenir[1], Bernardino Ghetti[3], Caroline Graff[4,5], Amit Kumar[4], Agneta Nordberg[4,5], Michel Goedert[1*], Sjors H.W. Scheres[1*]

[1]Medical Research Council Laboratory of Molecular Biology, Cambridge, UK
[2]RIKEN Brain Science Institute, Saitama, Japan
[3]Department of Pathology and Laboratory Medicine, Indiana University School of Medicine, Indianapolis, IN, USA
[4]Department of Neurobiology, Care Sciences and Society, Karolinska Institutet, Stockholm, Sweden
[5]Theme Inflammation and Aging, Karolinska University Hospital, Stockholm, Sweden

[±] Present address: Medical Research Council Prion Unit and Institute of Prion Diseases, University College London, London, UK
[☓] Present address: Dementia Research Institute, Department of Clinical Neurosciences, University of Cambridge, Cambridge, UK
[+] Present address: Department of Neurocognitive Science, Nagoya City University, Nagoya, Japan

*Corresponding authors: mg@mrc-lmb.cam.ac.uk and scheres@mrc-lmb.cam.ac.uk

SUPPLEMENTARY TABLE

Table S1. Cryo-EM data acquisition and structure determination.

	**Human Arctic**		**Mouse *App*$^{NL-G-F}$**
	**Tetramer**	**Dimer**	
	(EMD-16023, PDB 8BG0)	(EMD-16022, PDB 8BFZ)	(EMD-16027, PDB 8BG9)
**Data acquisition**			
Electron gun	XFEG		XFEG
Detector	K3		K3
Energy filter slit (eV)	20		20
Magnification	105,000		105,000
Voltage (kV)	300		300
Electron dose (e^-/Å^2)	40		40
Defocus range (μm)	0.8 to 2.4		1.0 to 2.6
Pixel size (Å)	0.653		0.86
**Map refinement**			
Symmetry imposed	C2	C2	C1
Initial particle images (no.)	279,809		238,564
Final particle images (no.)	115,228	34,191	39,823
Map resolution (Å)	2.0	2.8	3.5
FSC threshold	0.143	0.143	0.143
Helical twist (°)	-3.0	-2.9	179.3
Helical rise (Å)	4.7	4.8	2.46
**Model refinement**			
Model resolution (Å)	2.0	2.8	3.5
FSC threshold	0.5	0.5	0.5
Map sharpening *B* factor (Å^2)	-39	-56	-82
**Model composition**			
Non-hydrogen atoms	6496	1326	1692
Protein residues	896	186	222
Ligands	0	0	0
***B* factors (Å^2)**			
Protein	49	55	86
**R.m.s. deviations**			
Bond lengths (Å)	0.009	0.009	0.007
Bond angles (°)	1.5	1.5	1.5
**Validation**			
MolProbity score	1.4	2.2	3.1
Clashscore	1.1	3.3	12.1
Poor rotamers (%)	1.2	6.8	12.5
**Ramachandran plot**			
Favored (%)	92.3	93.1	82.9
Allowed (%)	7.7	6.9	17.1
Disallowed (%)	0	0	0

SUPPLEMENTARY FIGURES

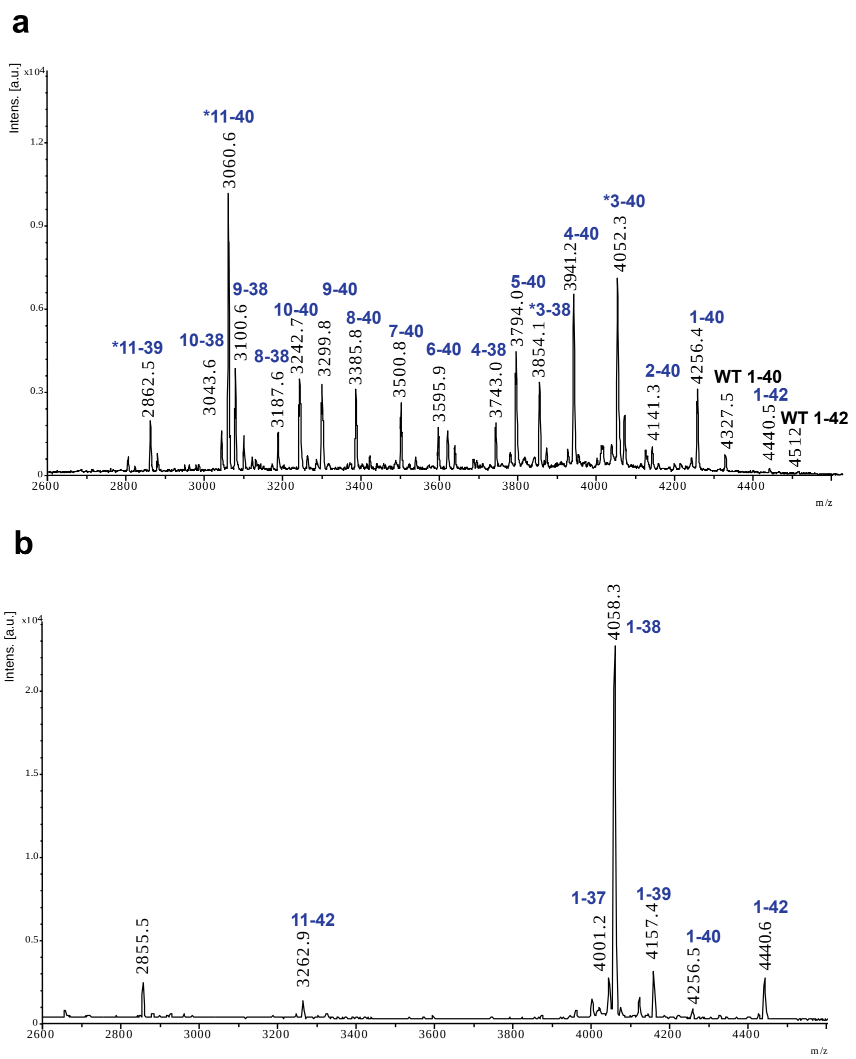

Figure S1 Mass spectrometric analysis.

a. MALDI mass spectra for Aβ from the sarkosyl-insoluble fractions used for cryo-EM of human E22G Aβ. Mutant Aβ (blue), wild-type Aβ (black). Starred blue peptides are pyroglutamate-modified.

b. MALDI mass spectra for Aβ from the sarkosyl-insoluble fractions used for cryo-EM of *App*$^{NL\text{-}G\text{-}F}$ mouse brains. Mutant Aβ in blue.

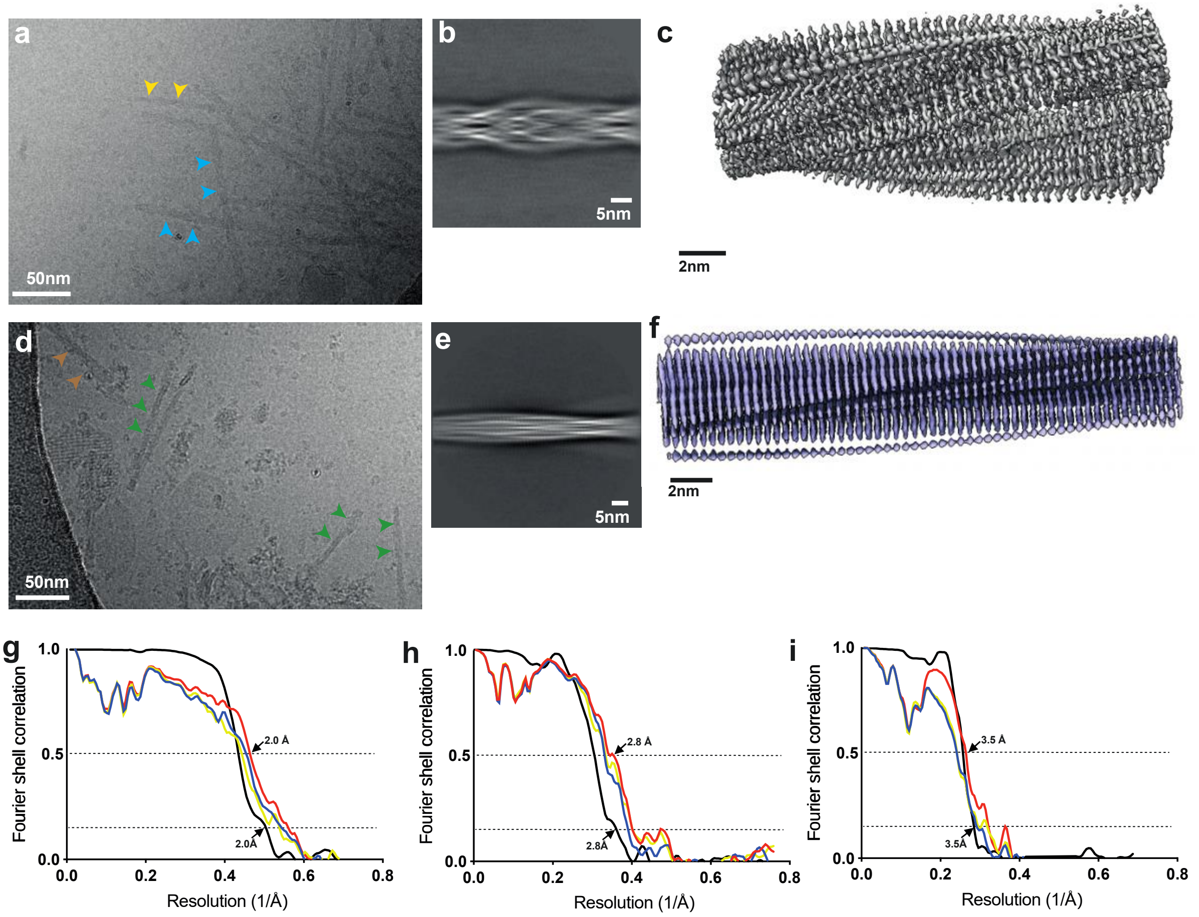

Figure S2 Cryo-EM micrographs and processing details.

a,d. Cryo-EM micrographs of E22G Aβ filaments from temporal cortex of case *AβPParc1* (a) and E22G Aβ filaments from *App*$^{NL\text{-}G\text{-}F}$ mouse brains (d). In (a), human Arctic tetrameric filaments are indicated with cyan arrows, dimeric type II Aβ42 filaments are indicated with orange arrows. In (d), dimeric filaments with the murine Arctic fold are indicated with green arrows and tetrameric filaments with the murine Arctic fold are indicated with brown arrows. Scale bar, 50 nm.

b,e. 2D class averages of E22G Aβ filaments from human brain (b) and E22G Aβ filaments from *App*$^{NL\text{-}G\text{-}F}$ mouse brains (e).

c,f. 3D reconstructions of E22G Aβ filaments from human brain (c) and E22G Aβ filaments from *App*$^{NL\text{-}G\text{-}F}$ mouse brains (f). Scale bar, 2 nm.

g,h,i. Fourier shell correlation (FSC) curves for cryo-EM maps and structures of human Arctic tetrameric filaments (g), human Arctic dimeric filaments (h) and mouse *App*$^{NL\text{-}G\text{-}F}$ Arctic dimeric filaments (i). FSC curves for two independently refined cryo-EM half maps are shown in black; for

the final refined atomic model against the final cryo-EM map in red; for the atomic model refined in the first half map against that half map in blue; and for the refined atomic model in the first half map against the other half map in yellow.

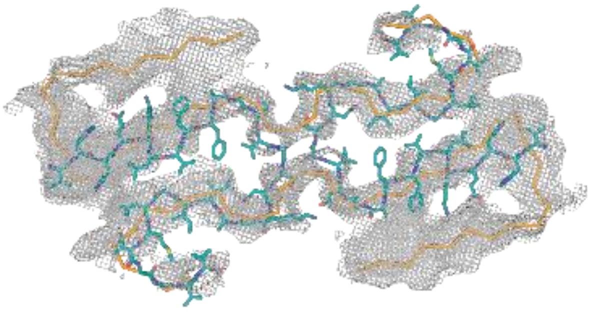

Figure S3 Comparison of an Arctic fold-based model of symmetrical Aβ40 doublet filaments with the structure of Aβ40 filaments from the meninges of Alzheimer's disease

The model of filaments comprising two identical protofilaments of Arctic fold A was built by taking one of the two A:B doublets shown in Figure 1b and replacing fold B with fold A, overlaid on the substructure common to both folds. This model, which was fitted into the 4.4 Å resolution density map of Aβ40 filaments from meninges (EMD:10204; grey mesh), is shown in full-atom representation (Arctic protofilament folds A in cyan). The structure that corresponds to this map is shown in orange (PDB:6SHS).
